# Spectroscopic and Microscopic Analyses of Fe_3_O_4_/Au Nanoparticles Obtained by Laser Ablation in Water

**DOI:** 10.3390/nano10010132

**Published:** 2020-01-10

**Authors:** Maurizio Muniz-Miranda, Francesco Muniz-Miranda, Emilia Giorgetti

**Affiliations:** 1 Department of Chemistry “Ugo Schiff”, University of Florence, Via Lastruccia 3, 50019 Sesto Fiorentino, Italy; 2 Institute of Complex Systems (CNR), Via Madonna del Piano 10, 50019 Sesto Fiorentino, Italy; emilia.giorgetti@fi.isc.cnr.it; 3 École Nationale Supérieure de Chimie de Paris and PSL Research University, CNRS, Institute of Chemistry for Life and Health Sciences (i-CLeHS), FRE 2027, 11, rue Pierre et Marie Curie, F-75005 Paris, France; f.muniz-miranda@chimieparistech.psl.eu

**Keywords:** laser ablation, gold, magnetite, SERS, 2,2′-bipyridine

## Abstract

Magneto-plasmonic nanoparticles constituted of gold and iron oxide were obtained in an aqueous environment by laser ablation of iron and gold targets in two successive steps. Gold nanoparticles are embedded in a mucilaginous matrix of iron oxide, which was identified as magnetite by both microscopic and spectroscopic analyses. The plasmonic properties of the obtained colloids, as well as their adsorption capability, were tested by surface-enhanced Raman scattering (SERS) spectroscopy using 2,2′-bipyridine as a probe molecule. DFT calculations allowed for obtaining information on the adsorption of the ligand molecules that strongly interact with positively charged surface active sites of the gold nanoparticles, thus providing efficient SERS enhancement. The presence of iron oxide gives the bimetallic colloid new possibilities of adsorption in addition to those inherent to gold nanoparticles, especially regarding organic pollutants and heavy metals, allowing to remove them from the aqueous environment by applying a magnetic field. Moreover, these nanoparticles, thanks to their low toxicity, are potentially useful not only in the field of sensors, but also for biomedical applications.

## 1. Introduction

Nanoparticles constituted of metals like silver, gold, or copper exhibit plasmonic properties and are widely employed as biosensors, drug vectors, and SERS (surface-enhanced Raman scattering) [1,2] and fluorescence markers, especially gold nanoparticles that are more biocompatible. Their applications can be realized by adding additional functionalities like magnetic properties. Hence, in nanomedicine they find diagnostic and/or therapeutic applications in magnetic resonance imaging or for generating hyperthermia by applying locally intense magnetic fields [3,4,5,6]. In this regard, Fe_3_O_4_ magnetic nanoparticles, presenting good biocompatibility and low toxicity [7,8,9], are widely used in these biomedical applications. Usually, different chemical procedures are employed to prepare these nanocomposites [10,11,12,13,14,15,16,17] to be used for sensoristic and biomedical applications, but they involve problems due to the presence of surfactants, stabilizers, residual reductants, and by-products, which could interfere in both the adsorption and the detection of ligands. In this regard, laser-assisted procedures have been recently employed to obtain metal nanoparticles with both plasmonic and magnetic properties [18,19,20]. In the past, some of us adopted the laser ablation procedure of metal targets in water to produce bimetallic colloidal nanoparticles [21,22]; in particular, two-step laser ablations of iron and silver [23] and of nickel and silver [24] were employed to obtain magneto-plasmonic colloidal nanoparticles.

Here, we propose the fabrication of bifunctional Fe_3_O_4_/Au nanoparticles obtained by the two-step laser ablation of iron and gold targets in water, along with microscopic and spectroscopic characterization. To this end, high-resolution transmission electron microscopy (HRTEM) and selected area electron diffraction (SAED) analyses have been performed, and visible absorption, XPS, Raman, and SERS spectra have been obtained. To obtain information on the type of ligand/metal adsorption provided by these nano-platforms, calculations based on density functional theory (DFT) have also been carried out using 2,2′-bipyridine as a molecular reporter.

The importance and novelty of the present investigation, in addition to producing “pure” colloidal suspensions—that is, without the aid of chemical reagents and surfactants—are due to the fact that gold nanoparticles are trapped in a ferromagnetic matrix, so they are preserved from colloidal collapse. In addition, it is possible to remove all the bimetallic material, including the possible load of adsorbed ligands, from the solvent and transport them thanks to the use of a magnetic field. In this regard, the presence of iron oxide gives the bimetallic colloid new possibilities for the adsorption of ligands, in addition to those inherent to gold nanoparticles, and also for removal of them from the aqueous environment, especially with regard to organic pollutants [25] and heavy metals [26]. Finally, our bimetallic colloids exhibit plasmonic properties, in addition to magnetic ones, due to the presence of gold nanoparticles, which allow application of the SERS technique for sensoristic purposes. In practice, SERS spectroscopy provides huge intensification of the Raman signal of molecules adsorbed on nanostructured gold or silver surfaces, usually up to 10^7^ enhancement factors with respect to the normal Raman response of non-adsorbed molecules.

## 2. Materials and Methods

### 2.1. Laser Ablation

Iron (Sigma-Aldrich, St. Louis, Missouri (USA), 99.99% purity) and gold (Goodfellow, Huntingdon (UK), 99.95% purity) plates were used as targets for the laser ablation. Colloidal suspensions were prepared by laser ablations of iron in deionized water (18.2 MX cm @ 25 C), and then of gold, by using the fundamental wavelength (1064 nm) of a Q-switched Nd:YAG laser (Quanta System G90-10: rep. rate 10 Hz, pulse width at FWHM of 10 ns). The laser pulse energy was set at 20 mJ/pulse, corresponding to 200 mW average power, focusing the laser light into a laser spot of approximately 1 mm diameter and corresponding fluence of 2.5 J/cm^2^. The target plate was fixed at the bottom of a glass vessel filled with 6 mL of liquid (height above the target: 2 cm). The irradiation time of the metal targets was about 20 min. To minimize effects due to crater formation in the metal targets, the glass vessel was manually rotated and translated, stopping the ablation process every three minutes. The laser pulse entered the vessel from above, thus impinging perpendicularly onto the target. These experimental procedures were chosen in order to obtain a valid colloidal stability, following the indications of our previous experiments [23].

### 2.2. UV–Visible Extinction Spectroscopy

UV–visible extinction spectra of the colloidal suspensions were obtained in the 200–800 nm region by using a Cary 5 Varian spectrophotometer (OPL (optical path length) = 2 mm). The observed bands were due to both absorption and scattering of the radiation.

### 2.3. Microscopic Techniques

TEM (transmission electron microscopy) and HRTEM (high-resolution TEM) images were obtained after dipping Ni grids in the colloidal suspensions. Microscopic measurements, EDX (energy-dispersive X-ray spectrometry) analysis, and SAED patterns were obtained using a Jeol 2010 instrument operating at 200 kV and equipped with an EDS Link ISIS EDX micro-analytic system.

### 2.4. Raman Spectroscopy

Raman spectra of the bimetallic nanoparticles deposited on aluminum plate were measured at different points of the dried sample by using a Renishaw RM2000 micro-Raman instrument equipped with a diode laser emitting at 785 nm. Sample irradiation was accomplished by using the 50× microscope objective of a Leica Microscope DMLM. The backscattered Raman signal was fed into the monochromator through 40 μm slits and detected by an air-cooled CCD (2.5 cm^−1^ per pixel) filtered by a double holographic Notch filters system. Spectra were calibrated with respect to a silicon wafer at 520 cm^−1^.

SERS spectra of 10^−4^ M 2,2′-bipyridine (Sigma-Aldrich, St. Louis, Missouri (USA), 99% purity) in bimetallic colloid were obtained after addition of 10^−2^ M NaCl (Sigma-Aldrich, St. Louis, Missouri (USA), 99.999% purity) in order to increase the SERS enhancement without compromising the colloidal stability. The 647.1 nm line of a Kripton ion laser and a Jobin-Yvon HG2S monochromator equipped with a cooled RCA-C31034A photomultiplier were used. A defocused laser beam with 100 mW power was employed for impairing thermal effects. Power density measurements were made using a power meter instrument (model 362; Scientech, Boulder, CO, USA) giving ∼5% accuracy in the 300–1000 nm spectral range.

### 2.5. X-ray Photoelectron Spectroscopy

XPS measurements were made using a non-monochromatic Mg Kα X-ray source (1253.6 eV) and a VSW HAC 5000 hemispherical electron energy analyzer operating in the constant pass energy mode at E_pas_ = 44 eV. The bimetallic colloidal samples were prepared just before the analysis by depositing a few drops of the colloidal suspensions on soda glass substrates and letting the solvent evaporate. In order to increase the amount of deposited nanoparticles, this procedure was repeated several times. Then, the glasses with bimetallic nanoparticles were introduced into the UHV system via a loadlock under inert gas (N_2_) flux and kept in the introduction chamber overnight, allowing the removal of volatile substances as confirmed by the achieved pressure value (2 × 10^−9^ mbar), just above the instrument base pressure. The obtained spectra were referenced to the C 1s core peak at 284.8 eV assigned to the adventitious carbon. The spectra were fitted using CasaXPS software version 2.3.15.

### 2.6. Density Functional Theory Calculations

All DFT calculations were carried out using the GAUSSIAN 09 package [27]. Optimized geometries were obtained at the DFT level of theory, employing the widely adopted Becke 3-parameter hybrid exchange functional (B3) combined with the Lee–Yang–Parr correlation functional (LYP) [28,29], along with the Lanl2DZ basis set and pseudopotential [30,31,32]. All parameters were allowed to relax and all calculations converged toward optimized geometries corresponding to energy minima, as revealed by the lack of negative values in the frequency calculation. Dispersion interactions were taken into account using Grimme’s D3 scheme along with Becke–Johnson damping [33]. A scaling factor of 0.98 for the calculated harmonic wavenumbers was employed, as usually performed in calculations at this level of theory [34,35,36,37,38]. The calculated Raman intensities were obtained by following the indications of reference [24].

## 3. Results and Discussion

### 3.1. Microscopic Investigation

The colloid obtained by laser ablation of an iron target in water has a zeta potential value of +20.0 mV, which is lowered to +13.9 mV when a gold target is also ablated. This lowering is due to the adsorption of negative ions deriving from the water environment on the (positive) surface of the gold nanoparticles. However, the zeta potential is sufficient to provide stability to the bimetallic colloid, with no precipitate visible a week after preparation. The zeta potential data are reported in Figure S1.

The bimetallic colloid presents a red color and exhibits magnetic properties, as shown in Figure S2. When approaching a magnet, the colloidal nanoparticles aggregate, until they appear as a dark red precipitate visible to the naked eye.

**Figure 1:**
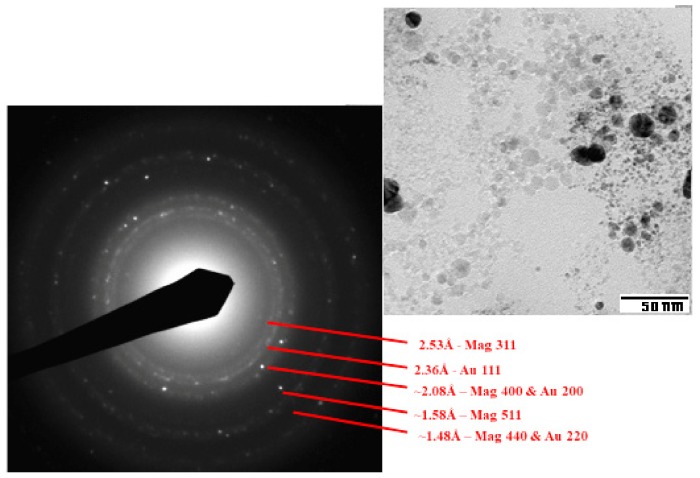
Low-magnification TEM micrograph of the Fe_3_O_4_/Au colloid (right), showing Au particles embedded in low-contrast matrix, along with SAED analysis (Mag: magnetite).

Observing the TEM images (see Figure 1), the colloid consists of spheroidal particles with dimensions ranging from a few nanometers to almost 20 nm in diameter. Based on the contrast, two kind of nanoparticles, with weaker contrast (low contrast, LC) and stronger contrast (high contrast, HC), can be distinguished. LC particles are mainly particles of a few nanometers, whereas the HC particles have two size classes: particles of a few nanometers and particles with a diameter of 10–20 nm. From the point of view of the metallic composition, the sample contains Fe and Au (in addition to O). The large HC particles are substantially composed of Au. Large LC particles are composed of Fe. The small particles, for which it is not possible to make EDX measurements on single individuals, show both Au and Fe. The EDX analyses of typical HC and LC nanoparticles are reported in Figure S3.

**Figure 2:**
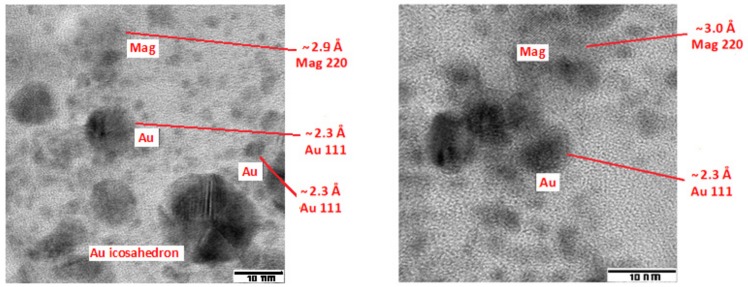
High-resolution TEM images of the Fe_3_O_4_/Au colloid, showing the interplanar distances in gold (Au) and magnetite (Mag) particles.

SAED on an enlarged field of the sample (Figure 1) shows interplanar distances consistent with magnetite (Fe_3_O_4_) and metallic gold. In particular, the ring around 2.36 Å is quite strong and must be attributed to the 111 reflection of gold [39]. Analysis of the high-resolution microscopic images (HRTEM) (Figure 2) shows interplanar distances typical of metallic Au for HC particles, both large and small. Crystalline growth in HC particles is observed as icosahedrons. LC particles show interplanar distances typical of magnetite [40], but also the presence of some small particles with amorphous characteristics. In conclusion, the gold nanoparticles appear to be embedded in a mucilaginous matrix consisting of magnetite in the form of nanoparticles of various sizes, with scarce tendency to aggregation, which can be separated from the aqueous environment under the action of a magnetic field.

### 3.2. Raman Spectra

**Figure 3:**
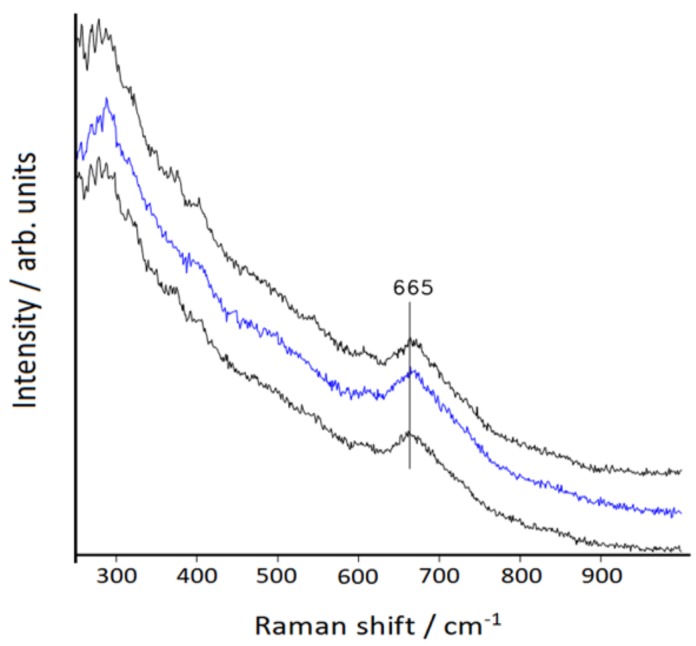
Micro-Raman spectra of the Fe_3_O_4_/Au nanoparticles deposited on Al plate at different points of the dry film. Excitation: 785 nm.

After centrifugation of a portion of the bimetallic colloid, the precipitate was examined using a micro-Raman spectrometer and showed the typical Raman band of magnetite (Fe_3_O_4_) at 665 cm^−1^ (see Figure 3), in agreement with the literature [41,42], confirming the magnetic properties of the nanosystem.

### 3.3. XPS Measurements

**Figure 4:**
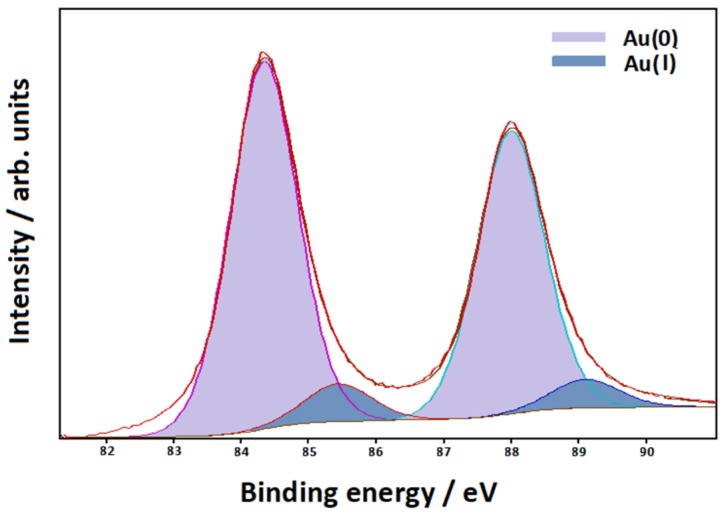
XPS spectrum of the bimetallic nanoparticles in the gold f_7/2_–f_5/2_ spectral region.

The XPS spectrum relative to the f_7/2_–f_5/2_ gold spectral region (see Figure 4) can be fitted by two components: the main one is located at 84.3 eV (f_7/2_), while the subordinate is located at a higher energy value (85.4 eV). These components can be attributed to Au(0) and Au(I), respectively, as well as occurring in the case of gold laser-ablated in deionized water [43].

### 3.4. UV–Visible Extinction Spectra

**Figure 5:**
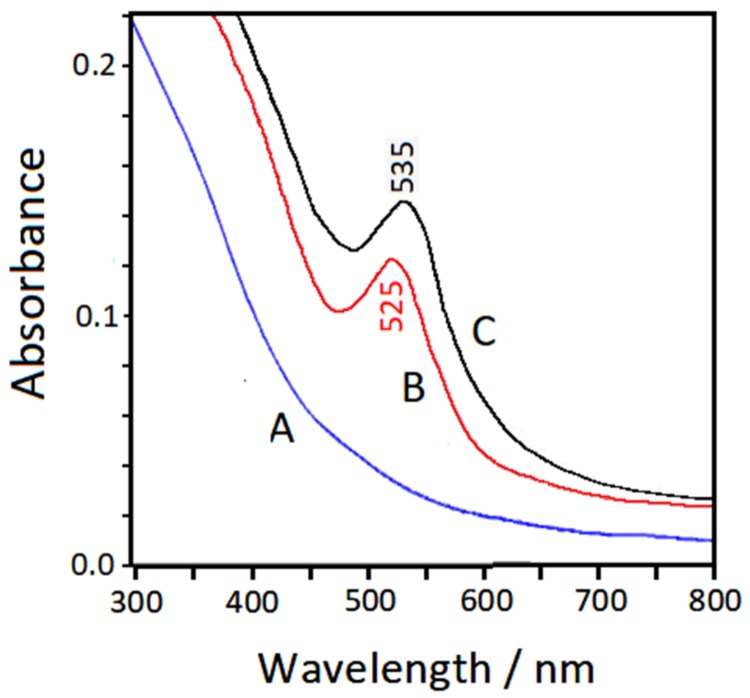
UV–visible extinction spectra of the Fe_3_O_4_ (A) and Fe_3_O_4_/Au (B) colloids. Spectrum C refers to the bimetallic colloid in the presence of 2,2′-bipyridine.

Figure 5 shows the UV–visible absorption spectra of the colloidal samples obtained by laser ablation of iron (Spectrum A) and then laser ablation of gold (Spectrum B). The band observed in Spectrum B around 525 nm is attributable to the surface plasmon resonance of non-aggregated gold nanoparticles. By adding 2,2′-bipyridine (bpy), the plasmon band is shifted to 535 nm.

### 3.5. Surface-Enhanced Raman Scattering

**Figure 6:**
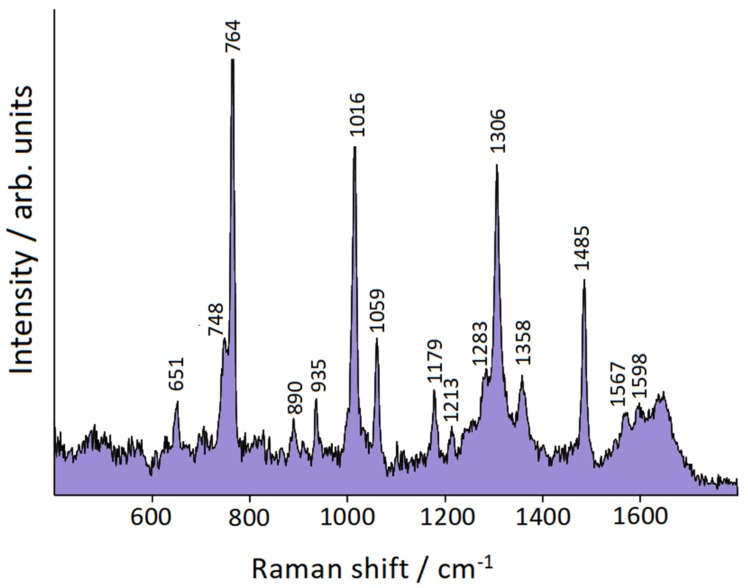
SERS spectrum of 2,2′-bipyridine in the bimetallic colloid. Excitation: 647.1 nm.

The evidence of the surface plasmon band of nanosized gold particles (see Figure 5) suggests the possibility of SERS activity of this bimetallic system. However, the molecular ligand needs to be effectively adsorbed to provide a reliable Raman enhancement. Hence, in order to find confirmation of our hypothesis, we checked the SERS response of the bimetallic colloid in the presence of 10^−4^ M 2,2′-bipyridine (bpy). By activation with NaCl, we observed a satisfactory SERS spectrum of bpy in the bimetallic colloid (Figure 6), with results quite similar to those reported in the literature for the adsorption of bpy on pure gold colloidal nanoparticles [44]; those frequencies are reported in Table 1 for comparison. This similarity indicates that our SERS spectrum is attributable to 2,2′-bipyridine bound to the gold nanoparticles present in the Fe_3_O_4_ colloidal matrix, which does not impair the ligand adsorption on gold. In Table 1 the IR and Raman frequencies of solid bpy [45] are also reported.

The addition of NaCl was necessary to obtain a satisfactory SERS spectrum of bpy. The presence of chloride anions, which strongly adsorb on the surface of the gold nanoparticles, has double validity because it can promote both the nanoparticle aggregation necessary for an efficient SERS response and the formation of active sites capable of strongly binding ligand molecules, similar to what occurs with silver nanoparticles activated by chloride anions [46,47,48]. In practice, in our bimetallic suspension it was not possible to obtain a valid SERS spectrum of 2,2′-bipyridine, even at 10^−4^ M concentration, unless we added NaCl. To induce particle aggregation or concentration, magnetic attraction could be employed, instead of adding chloride anions, in order to improve the SERS signal of the adsorbed ligands. In the future, this method will be tested by also evaluating the occurrence of possible problems in colloidal stability. In the present work, we used chloride activation to obtain an effective SERS response in a stable aqueous suspension in order to evaluate the possible use of these nanoparticles in biomedical applications.

However, one last problem remains to be solved: what kind of active site on the surface of the gold nanoparticles is involved in the interaction with the molecule, given that the XPS spectrum also showed the presence of ionized gold such as Au(I)? DFT calculations on the molecule linked to a neutral or a positively charged gold adatom can help in this purpose.

### 3.6. DFT Calculations

In Table 1 the experimental SERS frequencies of bpy are compared with those calculated for bpy/gold model complexes, along with the IR and Raman frequencies of solid bpy [45], whose molecules present a trans-planar structure. We observe that the prominent SERS bands (at 353, 651, 764, 1016, 1059, 1179, 1306, and 1485 cm^−1^) correspond to the bpy Raman bands of A_g_ symmetry species. For the simulation of the SERS spectra of the adsorbed bpy, we used the functional B3LYP, along with the Lanl2DZ basis set.

The choice to use this basis set was justified by the following considerations.

(a). This basis set has been widely employed in many literature articles to successfully reproduce both the structural and vibrational properties of different molecules. Here we report only a few very recent examples [49,50,51,52,53,54,55].
(b). Core electrons can be treated in an approximate way via effective core potentials (ECPs). This treatment includes scalar relativistic effects, which are important for the proper description of the geometric, electronic, and spectroscopic properties of heavy atoms. The LanL2DZ basis set is the best known basis set for molecular systems containing these atoms and for the efficient simulation of the Raman spectra of complexes with transition metals and the SERS spectra of molecules adsorbed on silver or gold nanoparticles, as demonstrated by many recent papers (for example, [38,50,52,53,54,55]).

We also tested the reliability of this basis set by examining the free 2,2′-bipyridine molecule in its typical *trans* conformation and comparing our DFT results with those reported in the literature [48] for the same molecule, with the same functional but with a different basis, 6-31+G*. The Lanl2DZ basis set used by us provided results generally comparable with those reported in the literature, as shown in the Supplementary Materials, regarding both structural parameters (Table S1) and vibrational frequencies (Table S2).

DFT calculations were performed for two gold complexes, where the bpy molecule in *cis* conformation is linked by means of the nitrogen atoms to a neutral Au atom or to a gold cation, Au^+^. The complex bpy/Au^+^ better reproduces the observed SERS frequencies than the complex bpy/Au°. In the first case, the average error between the calculated and observed frequencies is 7.75 cm^−1^; in the second one, the average error is significantly larger at 13.27 cm^−1^. In addition, the interaction of the molecule with a neutral atom is quite weak, in comparison with the interaction with Au^+^, as shown by the bpy→gold electronic charge transfers and the N–gold bond distances reported in Table 2, with |*e*| being the unsigned electron charge. The Mulliken partial charges are reported in Table S1. Hence, it is possible to conclude that the ligand molecules, when they adsorb on gold, strongly interact with positively charged active sites of the nanoparticle surface. Figure S4 shows the calculated normal modes of the bpy/Au^+^ complex relative to the prominent SERS bands. All these correspond to in-plane vibrations of the bpy molecule, in particular, the bands observed at 356, 651, 764, and 1016 cm^−1^ correspond to ring deformations, and those at 1306 and 1485 cm^−1^ to H bending modes.

To better quantify the charge transfer, we also employed a descriptor (called D_CT_, charge transfer distance) [56] that was mainly proposed to describe electron–hole displacement in optical excitations (S*_n_*→S_0_, *n* = 1, 2…, with S being singlet electronic states). The D_CT_ version adopted here is based on a partial charge (namely Mulliken’s) approach, using the spreadsheet reported in the Supplementary Materials of reference [56] and already employed with success for electronic transitions [37,57]. With the D_CT_ scheme, the difference between the electronic density of the ground state (S_0_) and the excited state of interest (S*_n_*) gives rise to a charge separation that can be modeled in a dipolar fashion due to a barycenter of reduced electronic charge (*Q+* here) and a barycenter of increased electronic charge (*Q*− here). The vector connecting the two points gives a straightforward depiction of the direction and magnitude of the overall charge movement and allows for calculating the amount of charge transferred. While this powerful yet easy approach was mainly developed to model different electronic states of the same system, it can, in principle, also be adopted for ground states of systems with different components (as long as the geometry of common moieties of the relaxed systems does not change significantly); this is discussed in more detail in the Supplementary Materials.

**Figure 7:**
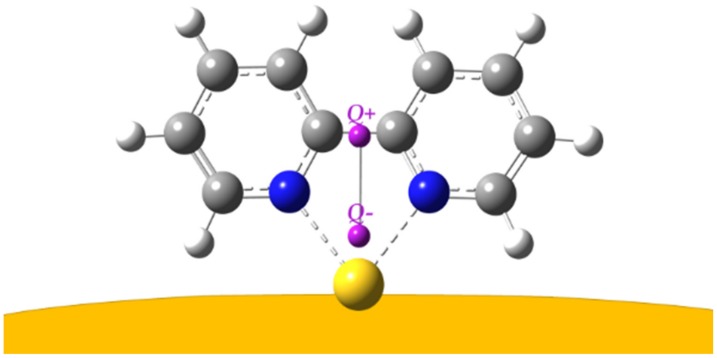
Adsorption model of bpy on Au^+^ adatom. Points *Q+* and *Q−* are the barycenters of the depletion and the increment of electron density, respectively, with respect to an isolated bpy molecule (*cis* conformation) and an isolated Au^+^ cation.

To the best of our knowledge, this is the first time the D_CT_ index has been adopted to describe charge rearrangements due to surface effects and not to light excitations, and it is reported in Figure 7.

With this approach, the computed charge transfer distance is about ~1.95 Å and the amount of charge moving is ~1.1 |*e*|, higher than that estimated from just the increase of electron charge on the Au atom; this is due to the fact that the D_CT_ takes into account the charge displacement over the whole system.

Finally, it is appropriate to define the limits of the DFT calculation model used by us, based on the chemical interaction between a bpy molecule and a single (positively charged) metal adatom. This complex correctly reproduces the positions of the SERS bands, because it is able to predict how the structure and, therefore, the force constants of the molecule change due to interaction with the metal. However, our model fails to satisfactorily reproduce the observed SERS intensities, as shown in the simulated SERS spectrum reported in Figure S5. Actually, in the case of 2,2′-bipyridine, which is linked to gold in a bidentate way by means of the lone pairs of the nitrogen atoms, our model cannot simulate the effect that the gold nanoparticles have on the polarizability of the adsorbed molecule and, therefore, on the intensities of the observed SERS spectrum.

## 4. Conclusions

Stable nanoparticles constituted of gold and iron oxide were obtained in an aqueous environment by means of laser ablation of Fe and Au targets in two successive steps, avoiding the presence of surfactants, stabilizers, residual reductants, and by-products which could interfere in both the adsorption and the detection of ligands. By using this technique, a mere mixture of two different metal colloids is not obtained, because gold nanoparticles are found to be embedded in the colloidal matrix of iron oxide. The latter was identified as magnetite by both microscopic and spectroscopic analyses. The plasmonic properties of the obtained colloidal nanosystem, as well as its capability of ligand adsorption, were tested by SERS spectroscopy using 2,2′-bipyridine (bpy) as a probe molecule. Thanks to the DFT calculations performed on model systems of gold/ligand complexes, it is possible to argue that positively charged active sites of the gold nanoparticles are responsible for the adsorption of ligand molecules when these approach the metal surface. In this way, strong interaction takes place between molecule and metal, with consequent efficient SERS enhancement, involving the charge transfer of one electron from the molecule to the metal.

Unlike the mixed Ag/Fe_3_O_4_ and Ag/NiO colloids previously prepared by two-step laser ablation [23,24], the present magneto-plasmonic nanoparticles are more biocompatible and are therefore potentially useful not only in the field of sensors, but also for biomedical applications. Our bimetallic colloidal suspensions are expected to have very low toxicity. Gold nanoparticles are known to be biocompatible and chemically stable, making them ideally suitable for biological applications [58]. Also, magnetite nanoparticles can exhibit low toxicity [59,60,61], which is closely dependent on the preparation method. In this respect, laser ablation in pure water represents the procedure of choice for the best biocompatibility properties.

Finally, a possible interpretation of the connection between Fe_3_O_4_ and Au nanoparticles can be proposed. In our sample, colloidal gold is intimately linked to the ferromagnetic material constituted of a mucilaginous matrix of small magnetite (Fe_3_O_4_) nanoparticles. Hence, all the bimetallic material can be completely separated by magnetic attraction from the aqueous environment wherein it is dispersed. In the literature [26], ultrafine Fe_3_O_4_ nanoparticles were employed to remove heavy metal ions from contaminated waters, thanks to their excellent adsorption performance. In a similar way, the magnetite nanoparticles obtained by laser ablation could act as adsorbents for the laser-ablated gold nanoparticles, forming a mixed bimetallic colloidal suspension. In fact, we verified by XPS measurements and DFT calculations that our gold particles have a positively charged surface. This could make them suitable to be captured by the magnetite nanoparticles, similarly to what happens with heavy metal ions.

## Figures and Tables

**Table 1:** Observed and calculated frequencies (cm^−1^).

Symmetry	bpy	bpy/Au	bpy/Au+	bpy/Au°	bpy/Au
Species [45]	IR/Raman [45]	SERS	Calc.	Calc.	SERS [44]
B_u_	1575		1603	1592	
A_g_	1589	1598	1598	1594	1586
B_u_	1550		1590	1581	
A_g_	1572	1567	1575	1570	1562
A_g_	1482	1485	1491	1483	1479
A_g_	1446		1469	1460	
B_u_	1450		1445	1433	
B_u_	1410		1429	1419	
B_u_	1265	1358	1324	1323	
B_u_	1250		1331	1298	
A_g_	1309	1306	1306	1313	1301
A_g_	1301	1283	1289	1286	
A_g_	1236		1294	1274	
B_u_	1140	1213	1206	1184	
A_g_	1146	1179	1190	1176	1173
B_u_	1085		1128	1113	
A_g_	1094		1114	1096	
B_u_	1065		1078	1077	
A_g_	1044	1059	1062	1049	1057
B_g_	------		1039	1032	
B_u_	1040		1038	1023	
A_u_	------		1033	979	
A_g_	994	1016	1007	1022	1010
B_u_	995		993	985	
A_u_	975		993	983	
B_g_	------		991	976	
B_g_	909	935	923	913	
B_u_	890		825	827	
B_g_	815	890	908	913	
A_u_	755		786	774	
A_g_	764	764	760	767	761
B_g_	742	748	746	754	
A_u_	740		747	756	
B_u_	655		658	656	
A_g_	614	651	653	636	646
B_u_	620		632	617	
B_g_	550		555	561	
A_u_	------		447	479	
A_g_	440		441	415	
B_g_	409		422	415	403
A_u_	------		405	380	
A_g_	332	356	353	327	353
B_g_	224		226	241	

**Table 2:** Calculated charge transfers and bond distances.

Model Complex	Bpy→Gold Charge Transfer	N–Gold Bond Distance
bpy/Au°	−0.232 |*e*|	2.62 Å
bpy/Au^+^	−0.502 |*e*|	2.23 Å

## Supplementary Materials

The Supplementary Materials are available online at https://www.mdpi.com/2079-4991/10/1/132/s1. Figure S1: Zeta potential data; Figure S2: Fe_3_O_4_/Au bimetallic colloidal sample, before and after application of magnetic field; Figure S3: EDX analysis; Figure S4: Calculated normal modes of the bpy/Au+ complex relative to the prominent SERS bands. The hydrogen atoms are omitted; Figure S5: Simulated SERS spectrum for the bpy/Au+ complex; Table S1: Mulliken partial charges in the bpy/gold complexes; Table S2: Observed and calculated vibrational frequencies (cm^−1^) of bpy; Table S3: Mulliken partial charges.

## References

[1] Schlücker S. (2011). Surface Enhanced Raman Spectroscopy: Analytical, Biophysical and Life Science Applications.

[2] Procházka M. (2016). Surface-Enhanced Raman Spectroscopy, Bioanalytical, Biomolecular and Medical Applications.

[3] Wang Y.-X.J. (2001). Super paramagnetic iron oxide based MRI contrast agents: Current status of clinical applications. Quant. Imaging Med. Surg..

[4] Cervadoro A., Giverso C., Pande R., Sarangi S., Preziosi L., Wosik J., Brazdeikis A., Decuzzi P. (2013). Design maps for the hyperthermic treatment of tumors with superparamagnetic nanoparticles. PLoS ONE.

[5] Zhang Y., Qian J., Wang D., Wang Y., He S. (2013). Multifunctional gold nanorods with ultrahigh, stability and tunability for in vivo fluorescence imaging, SERS detection, and photodynamic therapy. Angew. Chem. Int. Ed..

[6] Park Y.I., Kim H.M., Kim J.H., Moon K.C., Yoo B., Lee K.T., Lee N., Choi Y., Park W., Ling D. (2012). Theranostic probe based on lanthanide-doped nanoparticles for simultaneous in vivo dual-modal imaging and photodynamic therapy. Adv. Mater..

[7] Yathindranath V., Rebbouh L., Moore D.F., Miller D.W., van Lierop J., Hegmann T. (2011). A versatile method for the reductive, one-pot synthesis of bare, hydrophilic and hydrophobic magnetite nanoparticles. Adv. Funct. Mater..

[8] Li Z., Wei L., Gao M.Y., Lei H. (2005). One-pot reaction to synthesize magnetite biocompatible magnetite nanoparticles. Adv. Mater..

[9] Zhang Y., Kohler N., Zhang M. (2002). Surface modification of superparamagnetic magnetite nanoparticles and their intracellular uptake. Biomaterials.

[10] Qu H., Lai Y., Niu D., Sun S. (2013). Surface-enhanced Raman scattering from magneto-metal nanoparticle assemblies. Anal. Chim. Acta.

[11] Sun L., He J., An S., Zhang J., Ren D. (2013). Facile one-step synthesis of Ag@Fe_3_O_4_ core–shell nanospheres for reproducible SERS substrates. J. Mol. Struct..

[12] Bao Z.Y., Dai J., Lei D.Y., Wu Y. (2013). Maximizing surface-enhanced Raman scattering sensitivity of surfactant-free Ag-Fe_3_O_4_ nanocomposites through optimization of silver nanoparticle density and magnetic self-assembly. J. Appl. Phys..

[13] Sharma G., Jeevanandam P. (2013). A facile synthesis of multifunctional iron Oxide@Ag core-shell nanoparticles and their catalytic application. Eur. J. Inorg. Chem..

[14] Prucek R., Tuček J., Kilianová M., Panáček A., Kvítek L., Filip J., Kolář M., Tománková K., Zbořil R. (2011). The targeted antibacterial and antifungal properties of magnetic nanocomposite of iron oxide and silver nanoparticles. Biomaterials.

[15] Wang L., Wang L., Luo J., Fan Q., Suzuki M., Suzuki I.S., Engelhard M.H., Lin Y., Kim N., Wang J.Q. (2005). Monodispersed Core-Shell Fe_3_O_4_@Au Nanoparticles. J. Phys. Chem. B.

[16] Reguera J., Jimenez de Aberasturi D., Henriksen-Lacey M., Langer J., Espinosa A., Szczupak B., Wilhelm C., Liz-Marzan L.M. (2017). Janus plasmonic-magnetic gold-iron oxide nanoparticles as contrast agents for multimodal imaging. Nanoscale.

[17] Ovejero J.G., Morales I., de la Presa P., Mille N., Carrey J., Garcia M.A., Hernando A., Herrasti P. (2018). Hybrid nanoparticles for magnetic and plasmonic hyperthermia. Phys. Chem. Chem. Phys..

[18] Tymoczko A., Kamp M., Rehbock C., Kienle L., Cattaruzza E., Barcikowski S., Amendola V. (2019). One-step synthesis of Fe-Au core-shell magnetic-plasmonic nanoparticles driven by interface energy minimization. Nanoscale Horiz..

[19] Bertorelle F., Pinto M., Zappon R., Pilot R., Litti L., Fiameni S., Conti G., Gobbo M., Toffoli G., Colombatti M. (2018). Safe core-satellite magneto-plasmonic nanostructures for efficient targeting and photothermal treatment of tumor cells. Nanoscale.

[20] Pan S., Liu Z., Lu W. (2019). Synthesis of naked plasmonic/magetic Au/Fe_3_O_4_ nanostructures by plasmon-driven anti-replacement reaction. Nanotechnology.

[21] Muniz-Miranda M., Caporali S., Marsili P., Giorgetti E. (2015). Fabrication and characterization of Ag/Pd colloidal nanoparticles as stable platforms for SERS and catalytic applications. Mater. Chem. Phys..

[22] Giorgetti E., Marsili P., Canton P., Muniz-Miranda M., Caporali S., Giammanco F. (2013). Cu/Ag-based bifunctional nanoparticles obtained by one-pot laser-assisted galvanic replacement. J. Nanopart. Res..

[23] Muniz-Miranda M., Gellini C., Giorgetti E., Margheri G. (2017). Bifunctional Fe_3_O_4_/Ag nanoparticles obtained by two-step laser ablation in pure water. J. Colloid Interface Sci..

[24] Gellini C., Deepak F.L., Muniz-Miranda M., Caporali S., Muniz-Miranda F., Pedone A., Innocenti C., Sangregorio C. (2017). Magneto-plasmonic colloidal nanoparticles obtained by laser ablation of nickel and silver targets in water. J. Phys. Chem. C.

[25] Gao F. (2019). An Overview of Surface-Functionalized Magnetic Nanoparticles: Preparation and Application for Wastewater Treatment. ChemistrySelect.

[26] Fato F.P., Li D.-W., Zhao L.-J., Qiu K., Long Y.-T. (2019). Simultaneous Removal of Multiple Heavy Metal Ions from River Water Using Ultrafine Mesoporous Magnetite Nanoparticles. ACS Omega.

[27] Frisch M.J., Trucks G.W., Schlegel H.B., Scuseria G.E., Robb M.A., Cheeseman J.R., Scalmani G., Barone V., Petersson G.A., Nakatsuji H. (2009). Gaussian 09.

[28] Lee C., Yang W., Parr R.G. (1988). Development of the Colle-Salvetti correlation-energy formula into a functional of the electron density. Phys. Rev. B.

[29] Becke A.D. (1993). Density-functional thermochemistry. III. The role of exact exchange. J. Chem. Phys..

[30] Hay P.J., Wadt W.R. (1985). Ab initio effective core potentials for molecular calculations. Potentials for the transition metal atoms Sc to Hg. J. Chem. Phys..

[31] Wadt W.R., Hay P.J. (1985). Ab initio effective core potentials for molecular calculations. Potentials for main group elements Na to Bi. J. Chem. Phys..

[32] Hay P.J., Wadt W.R. (1985). Ab initio effective core potentials for molecular calculations. Potentials for K to Au including the outermost core orbitals. J. Chem. Phys..

[33] Grimme S., Ehrlich S., Goerigk L. (2011). Effect of the damping function in dispersion corrected density functional theory. J. Comp. Chem..

[34] Muniz-Miranda M., Pagliai M., Muniz-Miranda F., Schettino V. (2011). Raman and computational study of solvation and chemisorption of thiazole in silver hydrosol. Chem. Commun..

[35] Pagliai M., Muniz-Miranda F., Schettino V., Muniz-Miranda M. (2012). Competitive Solvation and Chemisorption in Silver Colloidal Suspensions. Prog. Colloid Polym. Sci..

[36] Muniz-Miranda M., Muniz-Miranda F., Caporali S. (2014). SERS and DFT study of copper surfaces coated with corrosion inhibitor. Beilstein J. Nanotechnol..

[37] Muniz-Miranda F., Pedone A., Muniz-Miranda M. (2018). Spectroscopic and DFT investigation on the photo-chemical properties of a push-pull chromophore: 4-Dimethylamino-4′-nitrostilbene. Spectrochim. Acta A.

[38] Gellini C., Muniz-Miranda F., Pedone A., Muniz-Miranda M. (2018). SERS active Ag-SiO_2_ nanoparticles obtained by laser ablation of silver in colloidal silica. Beilstein J. Nanotechnol..

[39] Gracia Pinilla M.Á., Villanueva M., Ramos-Delgado N.A., Melendrez M.F., Menchaca J.L. (2014). Au and Cu nanoparticles and clusters synthesized by pulsed laser ablation: Effects of polyethylenimine (PEI) coating. Dig. J. Nanomater. Biostruct..

[40] Nedkov I., Merodiiska T., Kolev S., Krezhov K., Niarchos D., Moraitakis E., Kusano Y., Takada J. (2002). Microstructure and Magnetic Behaviour of Nanosized Fe_3_O_4_ Powders and Poly crystalline Films. Monatsh. Chem..

[41] Shebanova O.N., Lazor P. (2003). Raman spectroscopic study of magnetite (FeFe_2_O_4_): A new assignment for the vibrational spectrum. J. Solid State Chem..

[42] Li H., Qin L., Feng Y., Hu L., Zhou C. (2015). Preparation and characterization of highly water-soluble magnetic Fe_3_O_4_ nanoparticles via surface double-layered self-assembly method of sodium alpha-olefin sulfonate. J. Magn. Magn. Mater..

[43] Sylvestre J.-P., Poulin S., Kabashin A.V., Sacher E., Meunier M., Luong J.H.T. (2004). Surface Chemistry of Gold Nanoparticles Produced by Laser Ablation in Aqueous Media. J. Phys. Chem. B.

[44] Joo S.-W. (2006). Adsorption of Bipyridine Compounds on Gold Nanoparticle Surfaces Investigated by UV-Vis Absorbance Spectroscopy and Surface Enhanced Raman Scattering. Spectrosc. Lett..

[45] Ould-Moussa L., Castella-Ventura M., Kassab E., Poizat O., Strommen D.P., Kincaid J.R. (2000). Ab initio and density functional study of the geometrical, electronic and vibrational properties of 2,2′-bipyridine. J. Raman Spectrosc..

[46] Sanchez-Cortes S., Garcia-Ramos J.V., Morcillo G., Tinti A. (1995). Morphological Study of Silver Colloids Employed in Surface Enhanced Raman Spectroscopy: Activation when Exciting in Visible and Near-Infrared Regions. J. Colloid Interface Sci..

[47] Giorgetti E., Marsili P., Giammanco F., Trigari S., Gellini C., Muniz-Miranda M. (2015). Ag nanoparticles obtained by pulsed laser ablation in water: Surface properties and SERS activity. J. Raman Spectrosc..

[48] Lopez-Tocón I., Valdivia S., Soto J., Otero J.C., Muniz-Miranda F., Menziani M.C., Muniz-Miranda M. (2019). A DFT Approach to the Surface-Enhanced Raman Scattering of 4-Cyanopyridine Adsorbed on Silver Nanoparticles. Nanomaterials.

[49] Basha M.T., Alghanmi R.M., Shehata M.R., Abdel-Rahman L.H. (2019). Synthesis, structural characterization, DFT calculations, biological investigation, molecular docking and DNA binding of Co(II), Ni(II) and Cu(II) nanosized Schiff base complexes bearing pyrimidine moiety. J. Mol. Struct..

[50] Fiori-Duarte A.T., Bergamini F.R.G., de Paiva R.E.F., Manzano C.M., Lustri W.R., Corbi P.P. (2019). A new palladium(II) complex with ibuprofen: Spectroscopic characterization, DFT studies, antibacterial activities and interaction with biomolecules. J. Mol. Struct..

[51] Khodashenas B., Ardjmand M., Baei M.S., Rad A.S., Khiyavi A.A. (2019). Gelatin–Gold Nanoparticles as an Ideal Candidate for Curcumin Drug Delivery: Experimental and DFT Studies. J. Inorg. Organomet. Polym..

[52] Sahan F., Kose M., Hepokur C., Karakas D., Kurtoglu M. (2019). New azo-azomethine-based transition metal complexes: Synthesis, spectroscopy, solid-state structure, density functional theory calculations and anticancer studies. Appl. Organomet. Chem..

[53] Hmida W.B., Jellali A., Abid H., Hamdi B., Naili H., Zouari R. (2019). Synthesis, crystal structure, vibrational studies, optical properties and DFT calculation of a new luminescent material based Cu (II). J. Mol. Struct..

[54] Maiti N., Malkar V.V., Mukherjee T., Kapoor S. (2018). Investigating the interaction of aminopolycarboxylic acid (APCA) ligands with silver nanoparticles: A Raman, surface-enhanced Raman and density functional theoretical study. J. Mol. Struct..

[55] Ricci M., Lofrumento C., Becucci M., Castellucci E.M. (2018). The Raman and SERS spectra of indigo and indigo-Ag_2_ complex: DFT calculation and comparison with experiment. Spectrochim. Acta A.

[56] Jacquemin D., Le Bahers T., Adamo C., Ciofini I. (2012). What is the “best” atomic charge model to describe through-space charge-transfer excitations?. Phys. Chem. Chem. Phys..

[57] Ciofini I., Le Bahers T., Adamo C., Odobel F., Jacquemin D. (2012). Through-Space Charge Transfer in Rod-Like Molecules: Lessons from Theory. J. Phys. Chem. C.

[58] Shukla R., Bansal V., Chaudhary M., Basu A., Bhonde R.R., Sastry M. (2005). Biocompatibility of gold nanoparticles and their endocytotic fate inside the cellular compartment: A microscopic overview. Langmuir.

[59] Souza D.M., Andrade A.L., Fabris J.D., Valério P., Góes A.M., Leite M.F., Domingues R.Z. (2008). Synthesis and in vitro evaluation of toxicity of silica-coated magnetite nanoparticles. J. Non-Cryst. Solids.

[60] Auffan M., Rose J., Wiesner M.R., Bottero J.-Y. (2009). Chemical stability of metallic nanoparticles: A parameter controlling their potential cellular toxicity in vitro. Environ. Pollut..

[61] Mahmoudi M., Simchi A., Milani A.S., Stroeve P. (2009). Cell toxicity of superparamagnetic iron oxide nanoparticles. J. Colloid Interface Sci..

