# Supplementary material for: Spectroscopic and Microscopic Analyses of Fe3O4/Au Nanoparticles Obtained by Laser Ablation in Water
Source: Nanomaterials (Basel). 2020 Jan 10;10(1):132. doi: 10.3390/nano10010132 (PMC7023500; doi:10.3390/nano10010132)
Supplement: Supplementary file 1 [file nanomaterials-10-00132-s001.pdf]

Supplementary Materials

Spectroscopic and microscopic analyses of Fe_3O_4/Au nanoparticles obtained by laser ablation in water

Maurizio Muniz-Miranda,[1,2,*] Francesco Muniz-Miranda,[3] Emilia Giorgetti [2]

[1] Department of Chemistry "Ugo Schiff", University of Florence, Via Lastruccia 3, 50019 Sesto Fiorentino, Italy; maurizio.muniz@unifi.it

[2] Institute of Complex Systems (CNR), Via Madonna del Piano 10, 50019 Sesto Fiorentino, Italy; emilia.giorgetti@fi.isc.cnr.it

[3] École Nationale Supérieure de Chimie de Paris and PSL Research University, CNRS, Institute of Chemistry for Life and Health Sciences (i-CLeHS), FRE 2027, 11, rue Pierre et Marie Curie, F-75005 Paris, France; f.muniz-miranda@chimieparistech.psl.eu

* Correspondence: maurizio.muniz@unifi.it

Sample Name: Fe_ns

Results

			Mean (mV)	Area (%)	Width (mV)
**Zeta Potential (mV):**	20.0	**Peak 1:**	20.0	100.0	3.63
**Zeta Deviation (mV):**	3.63	**Peak 2:**	0.00	0.0	0.00
**Conductivity (mS/cm):**	0.00662	**Peak 3:**	0.00	0.0	0.00

Result quality Good

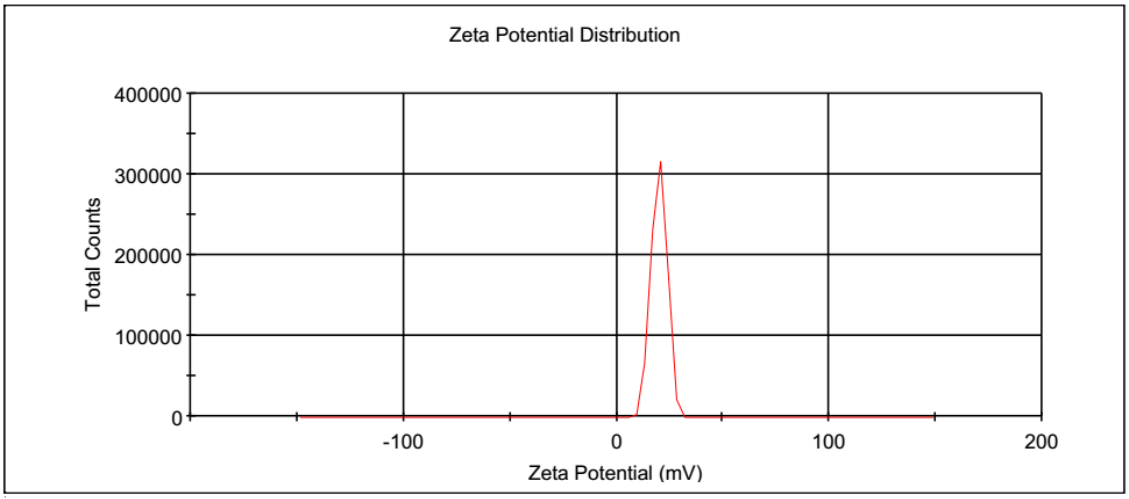

Sample Name: Au_ns -> Fe_ns

Results

			Mean (mV)	Area (%)	Width (mV)
**Zeta Potential (mV):**	13.9	**Peak 1:**	13.9	100.0	4.17
**Zeta Deviation (mV):**	4.17	**Peak 2:**	0.00	0.0	0.00
**Conductivity (mS/cm):**	0.0167	**Peak 3:**	0.00	0.0	0.00

Result quality Good

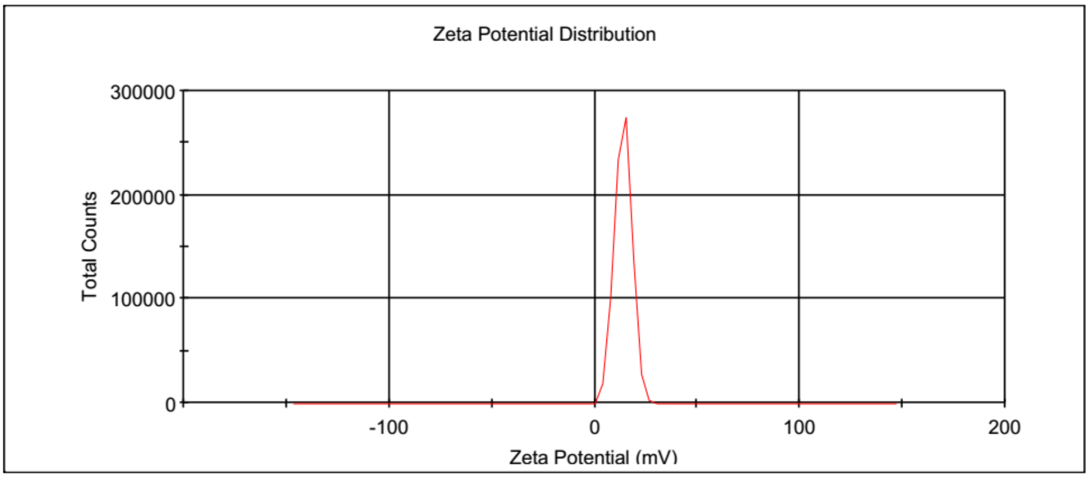

Fig. S1 – Zeta potential values for the colloid obtained by laser ablation of Fe (above) and for that obtained by two-step laser ablation of Fe and then of Au (below).

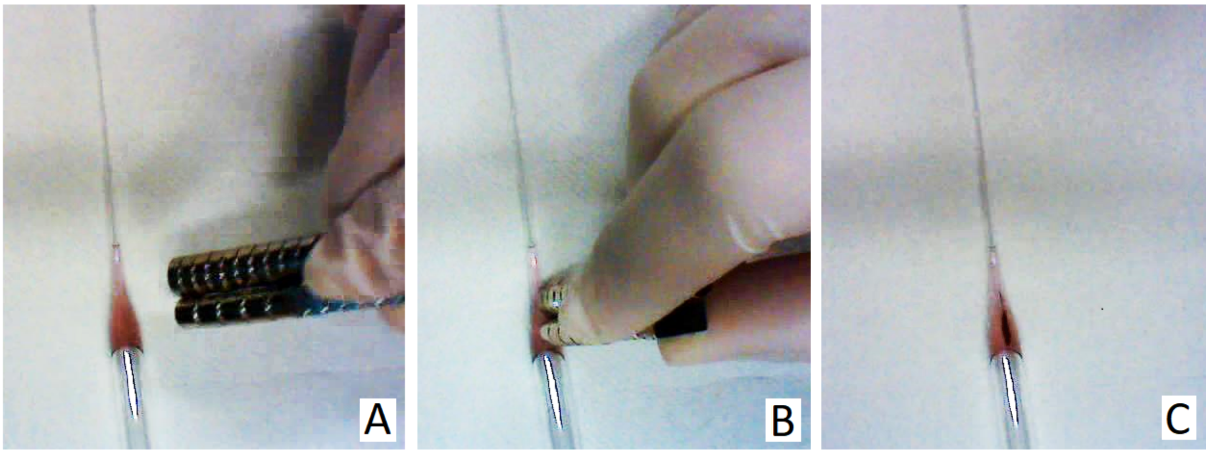

Fig. S2 – Fe_3O_4/Au bimetallic colloidal sample, as obtained by two-step laser ablation (A), after application of magnetic field (B) and after successive removing of the magnet (C).

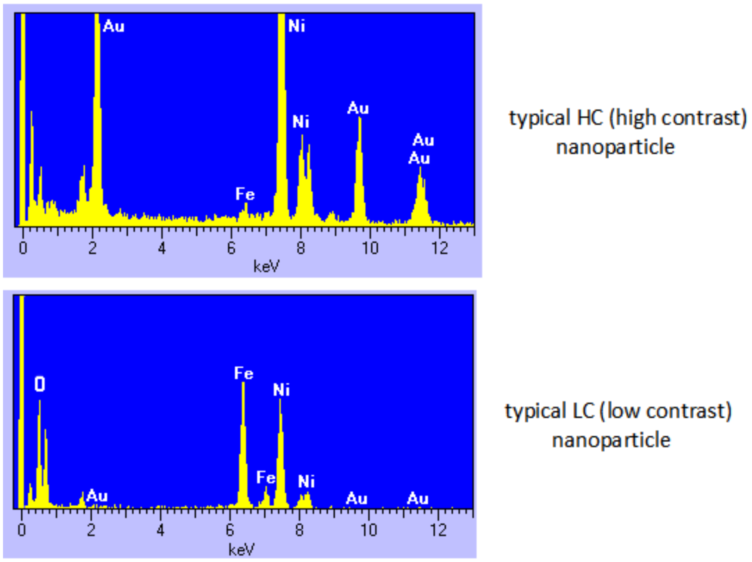

Fig. S3 - EDX analysis of HC and LC nanoparticles.

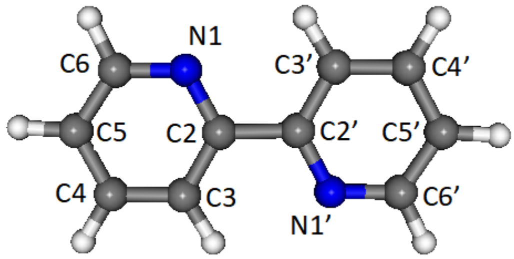

TABLE S1 – Structural parameters of bpy.

Bond distances/Å	Experimental data [a]	B3LYP/6-31G(+*) [b]	B3LYP/Lanl2dz [c]
N1C2	1.35	1.353	1.339
C2C3	1.41	1.406	1.398
C3C4	1.40	1.395	1.385
C4C5	1.37	1.398	1.389
C5C6	1.37	1.399	1.390
C6N1	1.37	1.343	1.329
C2C2'	1.50	1.490	1.489
Bond angles/°	Experimental data [a]	B3LYP/6-31G(+*) [b]	B3LYP/Lanl2dz [c]
N1C2C3	122.5	122.3	122.5
C2C3C4	118.3	119.0	118.9
C3C4C5	119.7	118.9	119.1
C4C5C6	118.5	118.1	118.5
C5C6N1	124.3	123.7	123.5
C6N1C2	116.7	117.9	118.5
N1C2C2'	116.1	116.8	117.1
C3C2C2'	121.4	120.9	120.9

[a] : Merritt. L.L. Jr, Schroeder E.D. *Acta Cryst*. **1956**, *9*, 801.

[b] : Ould-Moussa, L.; Castella-Ventura, M.; Kassab, E.; Poizat, O.; Strommen,D.P.; Kincaid, J.R. *J. Raman Spectrosc*. **2000**, *31*, 377–390.

[c] : present work.

TABLE S2 – Observed and calculated vibrational frequencies (cm^{-1}) of bpy.

Symmetry species	IR/Raman [a]	B3LYP/ 6-31G(+*) [a]	B3LYP/ Lanl2dz [b]
A_g	1589	1585	1597
B_u	1575	1582	1595
A_g	1572	1567	1582
B_u	1550	1554	1564
A_g	1482	1474	1487
A_g	1446	1445	1443
B_u	1450	1450	1454
B_u	1410	1423	1418
A_g	1309	1309	1326
A_g	1301	1299	1314
B_u	1265	1281	1308
B_u	1250	1254	1270
A_g	1236	1241	1274
B_u	1140	1167	1170
A_g	1146	1166	1170
B_u	1085	1091	1093
A_g	1094	1099	1100
B_u	1065	1066	1070
A_g	1044	1041	1042
B_g	------	1009	1038
B_u	1040	1034	1036
A_u	------	1008	1033
B_u	995	982	985
A_g	994	990	981
A_u	975	975	973
B_g	------	972	991
B_g	909	915	933
B_u	890	905	930
B_g	815	830	837
A_g	764	762	767
A_u	755	763	782
B_g	742	740	762
A_u	740	743	759
B_u	655	659	660
B_u	620	629	624
A_g	614	622	619
B_g	550	564	566
A_u	------	439	442
A_g	440	435	440
B_g	409	409	419
A_u	400	402	412
A_g	332	325	329
B_g	224	222	227

[a] : Ould-Moussa, L.; Castella-Ventura, M.; Kassab, E.; Poizat, O.; Strommen,D.P.; Kincaid, J.R. *J. Raman Spectrosc*. **2000**, *31*, 377–390.

[b] : present work.

TABLE S3 – Mulliken partial charges.

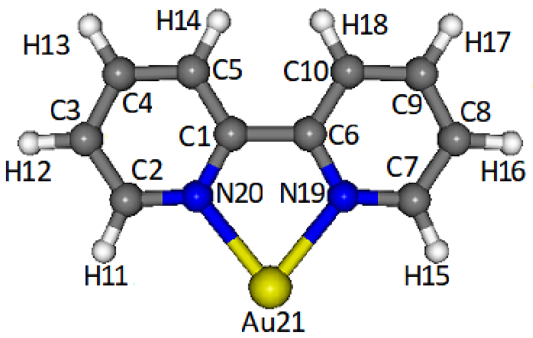

bpy/Au⁺ complex		bpy/Au° complex	
C1	0.2674	C1	0.1428
C2	-0.2083	C2	-0.2710
C3	-0.1387	C3	-0.1619
C4	-0.1678	C4	-0.1900
C5	-0.2392	C5	-0.2860
C6	0.2674	C6	0.1428
C7	-0.2083	C7	-0.2710
C8	-0.1387	C8	-0.1619
C9	-0.1678	C9	-0.1900
C10	-0.2392	C10	-0.2860
H11	0.2648	H11	0.2571
H12	0.2665	H12	0.2346
H13	0.2707	H13	0.2380
H14	0.2422	H14	0.2364
H15	0.2648	H15	0.2571
H16	0.2665	H16	0.2346
H17	0.2707	H17	0.2380
H18	0.2422	H18	0.2364
N19	-0.3064	N19	-0.0840
N20	-0.3064	N20	-0.0840
Au21	0.4979	Au21	-0.2323

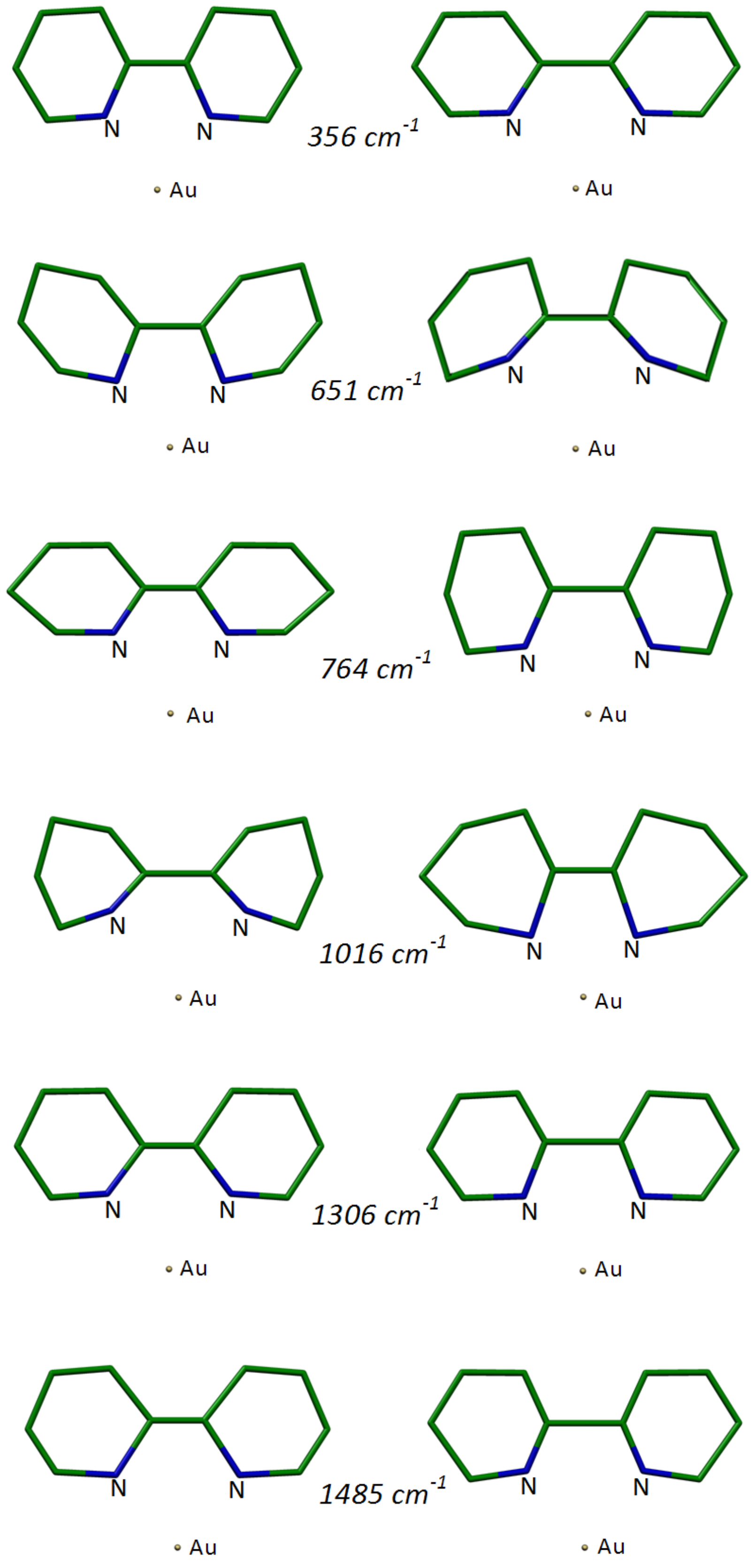

Fig. S4 – Calculated normal modes of the bpy/Au^+ complex relative to the prominent SERS bands. The hydrogen atoms are omitted.

Details on the D_{CT} calculations

The D_{CT} (charge-transfer distance) index was proposed to define a measure of the length of charge transfer (CT) excitation, as well as the amount of the transferred charge. This is achieved from the total electronic density computed for the ground and excited states, or adopting a suitable partition scheme for atomic charges (like Mulliken's).

However, the D_{CT} descriptor can be adopted also for ground states of systems with different components, as long as the geometry of common moieties of the relaxed systems do not change significantly.

In the case investigated in this paper, the previous statement is equivalent to say that the full Hamiltonian of the system (H_{total}, with total=bpy+gold) can be solved on the bases of the Hamiltonians of the subsystems (H_{bpy} and H_{gold}).

In practice, to compute the D_{CT} here we have followed this procedure:

1. we have obtained the relaxed geometry of the (bpy + Au^{+}) system;
2. for the starting state, we have given to bpy atoms the Mulliken charges of the isolated bpy and we have put a zero charge on the gold atom coordinates;
3. for the final state, we have given to the (bpy + Au^{+}) system their respective Mulliken charges;
4. finally, we have calculated the D_{CT} descriptor using the spreadsheet reported in the Supplementary Materials of ref. 56.

…………………………………………………………………………………………..

56. Jacquemin, D.; Le Bahers, T.; Adamo, C.; Ciofini, I. What is the ''best'' atomic charge model to describe through-space charge-transfer excitations? *Phys. Chem. Chem. Phys*. **2012**, *14*, 5383–5388.

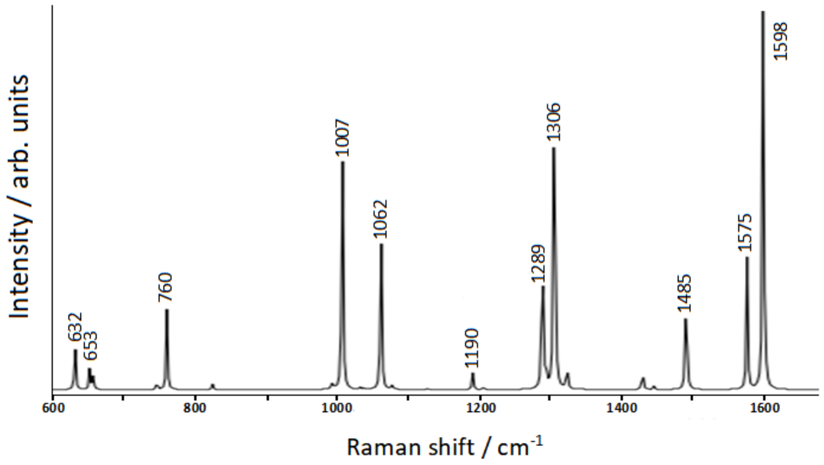

Fig. S5 – Simulated SERS spectrum for the bpy/Au$^+$ complex.
